# Ptomaines and Other Food Dangers

**Published:** 1907-01-26

**Authors:** 


### Ptomaines and Other Food Dangers.

The dangers which may be associated with the use of foods, improperly preserved or improperly prepared, have been conspicuously exhibited to the public view during the past week. First, the progress of a dispute in the courts of law, and the medical evidence given during its course, have suggested unpleasant possibilities in connection with so largely consumed an article of food as butchers' meat. The risks and dangers of canned meats have recently been brought home to everyone's mind. Now it is asked whether there may not be danger much closer at hand. Again, the statements made, on behalf of the deputation from the Infants' Health Society to the President of the Local Government Board, suggest that the very efforts made to guard against one set of dangers in connection with the milk supply, may, at times, introduce new and special dangers of their own. Altogether, therefore, it is not to be wondered at that the public mind should be somewhat disturbed by these revelations, and it important that the facts be placed in proper perspective.

With the particular issue raised in the case of alleged ptomaine poisoning we have here no concern, and it is not necessary for us even to allude to it. The possibility of such a form of poisoning has long been recognised by the medical profession, and its serious, and even fatal, consequences are well known. Further, it is a well-established observation, that the most severe manifestations of this form of poisoning have occurred from meat not obviously in a state of putrefaction. In these latter circumstances the recognition of an offensive smell is sufficient in itself to cause alarm, and to prevent the meat from being eaten. But even apart from this, there is good reason to believe that the more deadly products of proteid decomposition are those formed by the earlier chemical changes, and as these may exist without any offence to the sense of smell, there is an obvious lack of the protection which the later, and frankly putrefactive, changes afford. In saying this, however, it is necessary to reflect that experience shows the risk in any quantitative sense to be but a small one. Considering the large quantities of meat used, more particularly by the inhabitants of the British Isles, the rarity of well-established cases of ptomaine poisoning is a gratifying fact, and it reflects credit upon the great majority of those actively concerned with our meat supplies. This is not to say, by any means, that everything is perfectly satisfactory. Risks are run which ought not to be run, and the fact that they are often incurred with apparent immunity, is anything but a sufficient proof that the procedure is a wise one. To hear that each inspector in the great meat market at Smithfield has to deal with something like 200 tons a day, hardly conveys a conviction of security or suggests thoroughness and efficiency of method. And it may well be asked whether occasional cases of gastro-intestinal disturbance may not be due to moderate doses of ptomaines or such-like poisons. The supreme disaster of a fatal result forces public attention, but quite possibly much mischief is done short of the gravest of issues. Certainly there is need to insist strongly on the absolute duty of the authorities to see that food distributed to the public is pure and wholesome, and such a dispute as the one recently before the courts may have a good influence in this direction. Taking all the facts of the position into consideration, alarmist or sensational statements are entirely unwarranted. At the same time, it is well to recognise the possible mischiefs and to insist on the precautions by which alone such mischiefs can be prevented. Adequate and efficient inspection, and thorough cooking, are the two influences through which safety is to be secured, and whilst the private household may command the latter, the former is a responsibility which rests on the public authorities.

The milk supply and its dangers is another aspect of the food question constantly present to those concerned with the care of the public health, and this especially appeals to those interested in the subject of infant feeding. A few years ago great expectations were aroused by the proposal to institute milk depots, and to supply to the community milk which had been sterilised. Now, an authoritative medical deputation tells the President of the Local Government Board that sterilisation deprives milk of much of its nutritive value, and even renders it an unsuitable and undesirable food, at least for infants. There is certainly evidence in support of these conclusions, though we think the whole problem of excessive infantile mortality needs a wider and more comprehensive review than it has yet received. But granting the contention of the deputation, the story of milk sterilisation in relation to infant feeding is an admirable illustration of the essential complexity of food problems, and of the need of looking at such problems in the light of a full and exact experience. More than one confident scientific prediction in this particular field has been shown by practice to have been framed on a narrow and incomplete observation.

